# Effect of autologous platelet-rich plasma-releasate on intervertebral disc degeneration in the rabbit anular puncture model: a preclinical study

**DOI:** 10.1186/ar4084

**Published:** 2012-11-05

**Authors:** Shuji Obata, Koji Akeda, Takao Imanishi, Koichi Masuda, Won Bae, Ryo Morimoto, Yumiko Asanuma, Yuichi Kasai, Atsumasa Uchida, Akihiro Sudo

**Affiliations:** 1 Department of Orthopaedic Surgery, Mie University Graduate School of Medicine, 2-174 Edobashi, Tsu, 514-8507, Japan; 2 Department of Orthopaedic Surgery, University of California, San Diego, 9500 Gilman Drive, La Jolla, CA 92093-0863, USA; 3 Department of Radiology, University of California, San Diego, 408 Dickinson Street, San Diego, CA 92103-8226, USA; 4 Department of Spinal Surgery and Medical Engineering, Mie University Graduate School of Medicine, 2-174 Edobashi, Tsu, 514-8507, Japan

## Abstract

**Introduction:**

Platelet-rich plasma (PRP) is a fraction of plasma in which several growth factors are concentrated at high levels. The active soluble releasate isolated following platelet activation of PRP (PRP-releasate) has been demonstrated to stimulate the metabolism of IVD cells in vitro. The in vivo effect of PRP-releasate on degenerated IVD remains unknown. The purpose of this study was to determine the reparative effects of autologous PRP-releasate on degenerated intervertebral discs (IVDs).

**Methods:**

To induce disc degeneration, New Zealand white rabbits (n = 12) received anular puncture in two noncontiguous discs. Autologous PRP and PPP (platelet-poor plasma) were isolated from fresh blood using two centrifugation techniques. Four weeks after the initial puncture, releasate isolated from clotted PPP or PRP (PPP- or PRP-releasate), or phosphate-buffered saline (PBS; control) was injected into the punctured discs. Disc height, magnetic resonance imaging (MRI) T2-mapping and histology were assessed.

**Results:**

Anular puncture produced a consistent disc narrowing within four weeks. PRP-releasate induced a statistically significant restoration of disc height (PRP vs. PPP and PBS, *P*<0.05). In T2-quantification, the mean T2-values of the nucleus pulposus (NP) and anulus fibrosus (AF) of the discs were not significantly different among the three treatment groups. Histologically, the number of chondrocyte-like cells was significantly higher in the discs injected with PRP-releasate compared to that with PBS.

**Conclusions:**

The administration of active PRP-releasate induced a reparative effect on rabbit degenerated IVDs. The results of this study suggest that the use of autologous PRP-releasate is safe and can lead to a clinical application for IVD degeneration.

## Introduction

Intervertebral disc (IVD) degeneration is considered to be a multifactorial process involving mechanical, genetic, systemic, and biological factors. Biochemically, IVD degeneration is characterized by a change in extracellular matrix molecules (loss of proteoglycan and water content in the nucleus pulposus, or NP), resulting in an alteration of the biomechanical properties of IVD tissues. These degenerative changes are considered to induce the disruption (radial and circumferential tears, cracking, and fissuring) of IVD tissues, leading to degenerative disc diseases and, eventually, to low back pain [1-4]. Unfortunately, because of the absence of blood supply in the inner anulus fibrosus (AF) and NP, IVD tissues have little potential for self-repair. Thus, experimental treatment options for disc degeneration, encompassing molecular, gene, and cell therapies, are being actively pursued [5].

To develop biological therapies for IVD repair, molecular biology and tissue engineering technologies have recently been applied to improve the micro-environment of IVD tissues [6-14]. Growth factors are biologically active molecules capable of stimulating cellular growth, proliferation, and differentiation in an endocrine or paracrine fashion. Many growth factors, such as transforming growth factor-β (TGF-β), insulin-like growth factor-1 (IGF-1), basic fibroblast growth factor, platelet-derived growth factor (PDGF), epidermal growth factor (EGF), and bone morphogenetic protein (BMP)-2 and BMP-7 (otherwise known as osteogenic protein-1, or OP-1), have been shown to positively modulate the extracellular matrix of IVD cells (see review in [15]). Interestingly, in both *in vitro *and animal studies, autologous platelet-rich plasma (PRP), which contains concentrated levels of growth factors and other cytokines, has been shown to provide a 'local environment for tissue regeneration' [16]. Furthermore, the effective role of PRP on tissue repair or regeneration (or both) in a range of tissue types, including bone, cartilage, tendon, and muscle, has been reported (see review in [16]).

The principal function of platelets is to prevent bleeding. Platelets circulating in the blood rapidly adhere to damaged endothelial tissues in response to vessel injury. The activated platelets release plasma coagulation factors and adhesive protein, generating a fibrin clot. In addition, activated platelets release a range of growth factors (for example, IGF-1, TGF-β, PDGF, and EGF), cytokines (for example, interleukin-1β [IL-1β] and IL-8), and angiogenic factors (for example, vascular endothelial growth factor and angiopoietin-1) [17,18]. These biologically active molecules synergically promote hemostasis and tissue repair [17,19]. PRP is a plasma fraction that contains platelets concentrated at a high level. Because activated platelets have the potential to release growth factors, including IGF-1, TGF-β, PDGF, and EGF, PRP has been clinically used to accelerate wound healing and tissue regeneration in orthopedic, oral-maxillofacial, and plastic surgery [17,19,20]. Recently, the active soluble releasate isolated from PRP has been demonstrated to effectively stimulate the metabolism of articular chondrocytes [21] and IVD cells [22]*in vitro*. Therefore, we hypothesized that the administration of PRP-releasate into the degenerated IVD tissue could dramatically change the metabolic homeostasis and micro-environment within the IVD and finally improve the potential for self-repair. The purpose of this study was to determine the reparative effects of the *in vivo *administration of autologous PRP-releasate into degenerated IVDs in a well-established rabbit anular puncture model [23] using radiologic, magnetic resonance imaging (MRI) T2 mapping, and histological analyses.

## Materials and methods

### Animal surgery and blood sampling

A rabbit anular puncture model of disc degeneration was established, as previously reported [23]. Twelve female New Zealand white rabbits (Kitayama Labes Laboratory Animals Bleeding & Equipment Supply, Ina, Japan), ranging from 2.9 to 3.4 kg in body weight, were used in this study with the approval of our university's Institutional Animal Care and Use Committee. Preoperatively, rabbits were anesthetized by an intramuscular injection of ketamine hydrochloride (25 mg/kg; Ketalar; Sankyo Seiyaku, Tokyo, Japan) mixed with xylazine (5 mg/kg; Celactal; Bayer, Tokyo, Japan). Lateral plain radiographs were obtained to determine baseline IVD height values before puncture. Under general anesthesia with 3.0% isoflurane (Mylan Pharmaceutical Inc., Tampa, FL, USA), lumbar IVDs were exposed through a posterolateral approach, the initial puncture with an 18-gauge needle was performed on two non-contiguous discs (L2/3 and L4/5), and the disc (L3/4) between punctured discs was left intact as a control [11]. During surgery, 10 mL of fresh blood was drawn from the femoral artery with a 21-gauge needle into a syringe treated with 1 mL of anti-coagulant (citrate dextrose-A solution; Terumo, Tokyo, Japan). An additional 5 mL of fresh blood was drawn to isolate autologous serum to use in the activation step. The rabbits were returned to their cages after a short recovery observation and mobilized *ad libitum*.

### Preparation of platelet-rich plasma and platelet-poor plasma releasates

**Figure 1:**
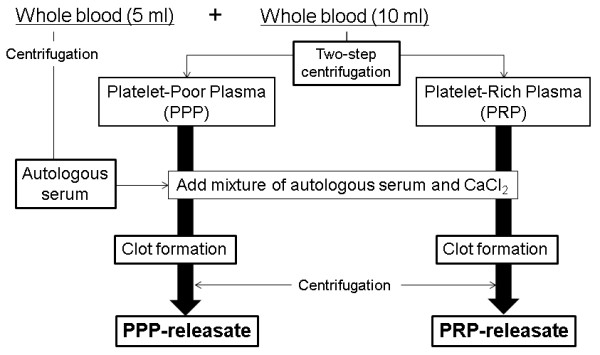
**Flowchart of the isolation of platelet-poor plasma (PPP) and platelet-rich plasma (PRP) releasates**. During surgery, a total of 15 mL of whole blood was drawn from the femoral artery. PPP and PRP were prepared by using two separate centrifugation techniques. Five milliliters of whole blood was used for isolating autologous serum. After clot formation by adding autologous serum and CaCl_2_, the PPP- and PRP-releasates were isolated from the clotted PPP and PRP by centrifugation.

PRP and PPP were prepared by using two centrifugation techniques (Figure 1). Whole blood collected by using an anti-coagulant was first centrifuged by using a centrifugation apparatus (Himac CT 6D; Hitachi Ltd., Tokyo, Japan) for 15 minutes at 330*g *to separate plasma and hemocyte fractions. The plasma fraction was then centrifuged for an additional 10 minutes at 1,000*g*. The supernatant plasma (PPP) was carefully removed, and the remaining PPP (approximately 200 μL) and precipitated platelets were designated as PRP. The number of platelets of each whole blood and PRP fraction was counted by using a hemocytometer. Autologous serum was prepared from the coagulated whole blood, which was centrifuged for 10 minutes at 1,000*g*. To activate platelets, PRP and PPP were treated with a mixture of autologous serum and 2% CaCl_2 _(Otsuka Pharmaceutical, Tokyo, Japan) for clot formation. The clotted PPP and PRP were allowed to rest at room temperature for more than 30 minutes, followed by centrifugation at 1,000*g *for 10 minutes. The resulting soluble releasate from the clot preparation of PRP (PRP-releasate) and PPP (PPP-releasate) was isolated and kept at -80°C until used.

### Experimental groups

Four weeks after the initial puncture, phosphate-buffered saline (PBS) (control) or PPP- or PRP-releasate was injected into the punctured discs through a contralateral side approach. The anterolateral surfaces of the previously punctured discs (L2/3-L4/5) were exposed with confirmation of osteophyte formation. Twelve rabbits were divided into two groups. One group (n = 4) received an injection of PBS (20 μL) at L2/3 and L4/5 discs into the center of the NP by using a 30-gauge needle. For the remaining rabbits (n = 8), each rabbit received one injection of PRP (20 μL) and one injection of PPP (20 μL) randomly given between L2/3 and L4/5. All preoperative and postoperative care was performed as in the initial operation.

### Radiographic analysis of disc height

Lateral plain radiographs of the lumbar spine were taken after administration of ketamine hydrochloride (25 mg/kg) and xylazine (5 mg/kg) at 2-week intervals up to 12 weeks after the initial puncture. Extreme care was taken to maintain a consistent level of anesthesia during radiography of each animal and at each time to obtain a similar degree of muscle relaxation, which may affect the disc height. All radiographic images were independently analyzed by using OsiriX Medical Image software (OsiriX Foundation, Geneva, Switzerland) by an orthopedic researcher who was blinded to the treatment groups. Data are reported as the IVD height expressed as the disc height index (DHI) (DHI = IVD height/adjacent vertebral disc height) [11,23]. Change in the DHI of injected discs was expressed as percentage DHI (% DHI) and normalized to the measured preoperative IVD height: % DHI = (postoperative DHI/preoperative DHI) × 100 [11]. Non-responsive treated discs (which did not exhibit degenerated changes at 4 weeks) were excluded from the analysis.

### Magnetic resonance imaging T2 quantification

Twelve weeks after the initial puncture, all rabbits were sacrificed and the spinal columns (L1 to L6 vertebra) with surrounding soft tissues were isolated and subjected to quantitative T2 MRI analysis. MRI was performed by using a 3.0-Tesla imager (Achieva 3.0T; Philips, Amsterdam, The Netherlands) with a 3-inch birdcage extremity coil (Philips). Quantitative T2 mapping was performed by using a multi-echo spin-echo sequence in the sagittal plane. Scanning parameters were the following: time-to-repeat (TR) = 3,000 ms, time-to-echo (TE) = 20, 40, 400 ms (20 TEs), field of view = 10 cm, slice thickness = 2 mm, image matrix = 560 × 560, and number of excitation = 1. Total scanning time per sample was 10 minutes 39 seconds. For creating color-coded T2 maps, MRI images at multiple TE were imported into OsiriX Medical Image software with T2 mapping plug-in.

The regions of interest (ROIs) for the NP were defined on T2-weighted images taken at a TE of 100 ms, whereas the ROIs for the AF were defined on proton density-weighted images taken at a TE of 20 ms. These images were selected to best visualize the borders of the AF and NP. Signal intensity values within each ROI were averaged and then fit mono-exponentially to a T2 decay equation. To minimize the differences between animals, the T2 values of each experimental disc were normalized by that of the non-punctured disc (L3/4). Non-responsive treated discs (which did not exhibit degenerated MR morphology) were excluded from the analysis (PBS group; n = 7 discs, PPP group; n = 7 discs, PRP group; n = 8 discs).

### Histological examination

After MRI assessment, the experimental IVDs were excised from the vertebral body-disc-vertebral body unit, and each IVD was fixed in 4% paraformaldehyde for 7 days at 4°C and then decalcified in 0.5 M ethylenediaminetetraacetic acid (EDTA), embedded in paraffin, sectioned, and assessed by conventional histology. Mid-sagittal sections (5 μm) of each IVD were stained with either hematoxylin and eosin or with safranin-O. Blinded to the experiment, an observer analyzed the histological sections and graded them by using the recently established histology grading protocol [24].

The chondrocyte-like cells appeared as large cells encircled with pericellular matrix densely stained with safranin-O. The number of chondrocyte-like cells (more than 2.0 μm in diameter including pericellular matrix) in the serial sections (100 μm in width from upper to lower endplate) of the inner anterior and posterior AF, and the center of the NP was quantified by manually counting the cells by using a light microscope.

### Statistical analysis

The significance of differences among means of data on radiograph measurements was analyzed by two-way repeated measures analysis of variance (ANOVA). T2 values in MRI and the number of chondrocyte-like cells were analyzed by one-way ANOVA and Fisher's protected least significant difference as a *post hoc *test. The data are expressed as the mean ± standard error of the mean. Histology grading was analyzed by the Kruskal-Wallis test and Mann-Whitney *U *test for the effect of treatment. Statistical analysis was performed by using the StatView program (version 5.0; SPSS Inc., Chicago, IL) with a significance level of *P *of less than 0.05.

## Results

### Platelet count

The mean platelet count of PRP was about 29 times greater than that of whole blood (whole blood: 195.2 ± 6.4 × 10^3^/μL, PRP: 5,752.5 ± 393.7 × 10^3^/μL, *P *<0.01 versus whole blood).

### Radiographic assessment

**Figure 2:**
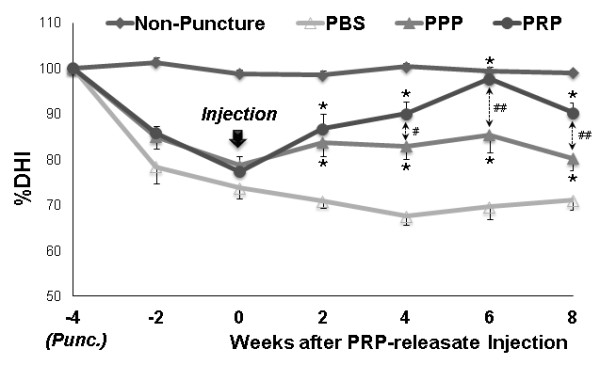
**Changes in the intervertebral disc height index (DHI)**. Four weeks after anular puncture [Punc. (at -4 weeks)], phosphate-buffered saline (PBS) (control) or platelet-poor plasma (PPP)- or platelet-rich plasma (PRP)-releasate was injected into the punctured discs. The percentage DHI was measured at each time point to quantify changes in disc height. Four weeks after the injection of PRP-releasate, the percentage DHI of injected discs in the PRP group was significantly higher than that in the PBS group (*P *<0.01, repeated analysis of variance). This significant difference in the percentage DHI was sustained until 8 weeks after the injection. Values are shown as mean ± standard error of the mean. *P <0.05, **P <0.01 versus PBS group; ^#^*P *<0.05, ^##^*P *<0.01 between PPP and PRP.

In all PBS, PPP, and PRP groups, radiographs 4 weeks after the initial anular puncture showed a significant narrowing of disc height compared with that of the non-punctured L3/4 disc (approximately 24.0% decrease compared with the baseline percentage DHI values before anular puncture, *P *<0.01) (Figure 2, see arrow). There were no significant differences in the percentage DHI among the experimental groups.

The treatment significantly affected the post-injection disc height (two-way repeated ANOVA, *P *<0.001). Two weeks after the injection of PPP- or PRP-releasate, the disc height began to recover compared with the discs injected with PBS (% DHI: PBS group: 70.8% ± 1.5%; PPP group: 83.7% ± 3.0%, *P *<0.01; PRP group: 86.8% ± 9.3%, *P *<0.01 all versus PBS group) (Figure 2). Four weeks after the injection, PRP-releasate induced restoration of disc height to the level approaching 90% of the non-punctured disc; this was sustained for the entire experimental period (4W, 6W, 8W: *P *<0.01 versus PBS group; 4W: *P *<0.05, 6W, 8W: *P *<0.01 versus PPP group) (Figure 2). While the PPP group also showed a significant recovery of disc height compared with the PBS group, it was no more than 80% of that of the non-punctured disc (4W, 6W, 8W: *P *<0.01 versus PBS group) (Figure 2). The injection of PBS did not induce restoration of disc height over the same time period (Figure 2).

### Magnetic resonance imaging assessment

**Figure 3:**
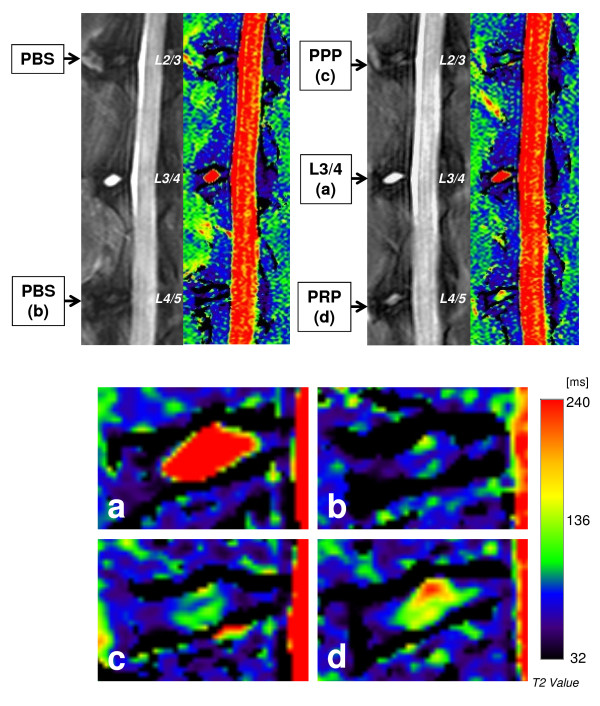
**Representative sagittal magnetic resonance T2-weighted images (left panels) and T2 maps (right panels) of rabbit lumbar spines**. The lower four images are magnifications (4×) of those discs in the upper two T2 maps: **(a) **non-punctured control (L3/4), **(b) **phosphate-buffered saline (PBS) (control) group, **(c) **platelet-poor plasma (PPP)-releasate group, and **(d) **platelet-rich plasma (PRP)-releasate group. The T2 values in the nucleus pulposus (NP) are significantly lower in all punctured discs (b-d) compared with non-punctured discs (a). However, areas with high T2 values area are identified in the center of the NPs injected with (c) PPP- and (d) PRP-releasates.

**Figure 4:**
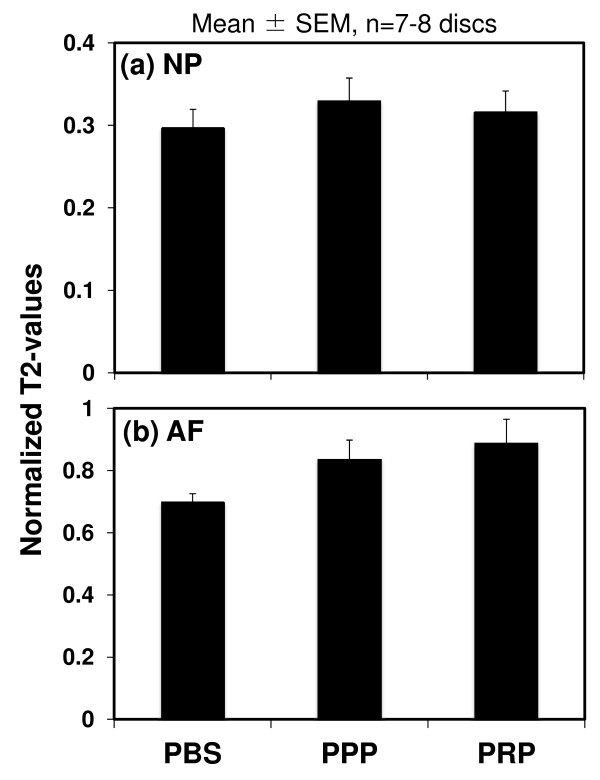
**Magnetic resonance imaging results of normalized T2 values**. Magnetic resonance imaging (MRI) analysis was performed at 8 weeks after the phosphate-buffered saline (PBS) (control), platelet-poor plasma (PPP)-releasate, and platelet-rich plasma (PRP)-releasate injections. The normalized T2 value of the PBS group was the lowest on average, but no statistical significance was found. Values are shown as mean ± standard error of the mean (SEM): PBS; n = 7 discs, PPP; n = 7 discs, PRP; n = 8 discs.

MRI analysis was performed at 8 weeks after the PBS, PPP, or PRP injections. Representative sagittal images of color-coded T2 maps showed that an area with high T2 value was identified in the NP injected with PRP-releasate (Figure 3). The normalized T2 values of the NPs (Figure 4a) were slightly higher than those for PPP (0.32 ± 0.03) and PRP (0.30 ± 0.02) samples compared with the values in the PBS samples (0.28 ± 0.02) but failed to reach statistical significance (*P *= 0.66). The normalized T2 values of the AFs (Figure 4b) showed similar trends, being somewhat higher for PRP (0.89 ± 0.08) and PPP (0.84 ± 0.06) compared with PBS control (0.70 ± 0.03). However, no statistically significant treatment effect was found (*P *= 0.49).

### Histological evaluations

**Figure 5:**
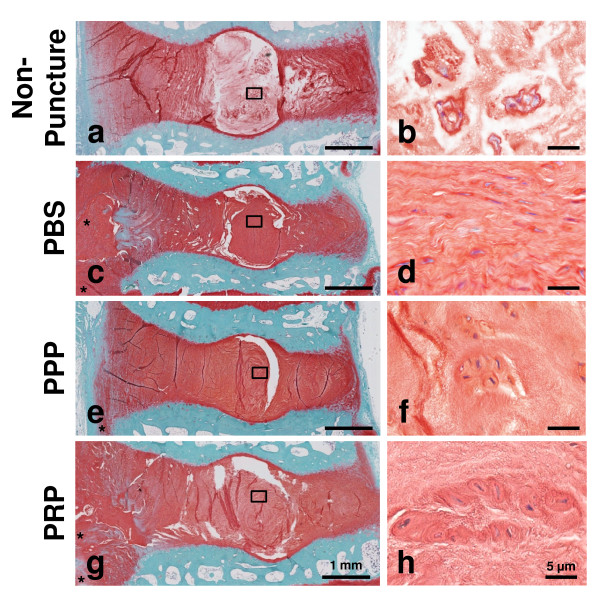
**Typical histological changes after injections of phosphate-buffered saline (PBS) (c,d), platelet-poor plasma (PPP)-releasate (e,f), or platelet-rich plasma (PRP)-releasate (g,h)**. On safranin-O-stained sections, a moderate-to-severe degeneration was observed in the PBS (c,d) and PPP (e,f) groups. In the PRP group, the increasing number of chondrocyte-like cells which appeared as large cells encircled with pericellular matrix densely stained with safranin-O were found in the nucleus pulposus (h). The histology of the non-punctured control (L3/4) is shown in (a,b). (b,d,f,h) are magnification images of the area indicated by a square (a,c,e,g), respectively. Scale bars: 1 mm (a,c,e,g) and 5 μm (b,d,f,h). Asterisk indicates osteophyte formation.

Safranin-O-stained histology in the PBS group (Figure 5c,d) showed severely degenerated discs in which most of the NP contents have been lost and collapsed, wavy fibrocartilage lamella and associated fibrochondrocyte-like cells of the AF. In the PPP and PRP groups, the increasing numbers of chondrocyte-like cells, which appear as large cells encircled with pericellular matrix densely stained with safranin-O, were found in either the inner AF or the NP (Figure 5f,h, respectively). However, there were no significant differences in the histological scores among the three groups. In all of the experimental groups, the invasion of blood vessels or inflammatory cells was not observed within the discs (Figure 5c,d, Figure 5e,f, and Figure 5g,h, respectively). Furthermore, no ossification of exclusively IVD tissues was found in any of the samples, although there was significant osteophyte formation at the edge of the vertebral body (Figure 5c,e,g).

**Figure 6:**
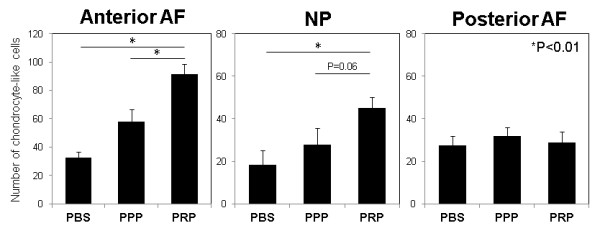
**The number of chondrocyte-like cells after injections of phosphate-buffered saline (PBS) (control), platelet-poor plasma (PPP)-releasate, or platelet-rich plasma (PRP)-releasate**. The numbers of chondrocyte-like cells in the inner anterior and posterior anulus fibrosus (AF) and the center of the nucleus pulposus (NP) were manually counted by using light microscopy. In the anterior AF, the number of chondrocyte-like cells in the PRP group was significantly higher than those of the PBS and PPP groups (*P *<0.01). The injection of PRP-releasate significantly increased the number of chondrocyte-like cells in the NP compared with that of the PBS group (*P *<0.01). Values are shown as mean ± standard error of the mean.

Within the present study, one of the major cellular changes was the increasing number of chondrocyte-like cells in the inner AF or in the NP of PRP-releasate-injected discs, as previously reported [24]. In the anterior (inner) AF, the number of chondrocyte-like cells in the PRP group was significantly higher than those of the PBS and PPP groups (*P *<0.01) (Figure 5). The injection of the PRP-releasate significantly increased the number of chondrocyte-like cells in the NP compared with that of the PBS group (*P *<0.01), although no significant differences were found between the PRP and PPP groups (*P *= 0.06) (Figure 6). In the posterior (inner) AF, no significant differences were found among the three groups (Figure 6).

## Discussion

This study examined the efficacy of the injection of PRP-releasate into degenerated discs in the well-established rabbit anular needle puncture model. The results of this study demonstrated that the intradiscal injection of PRP-releasate is effective for restoring disc height in this animal model. The histological analysis also revealed a reparative effect of PRP-releasate on the degenerated IVD.

Platelets have three major granule storage compartments: alpha granules, dense granules, and lysosomes. Alpha granules contain a large number of different secretory proteins, such as growth factors, coagulation proteins, adhesion molecules, cytokines, cell-activating agents, and angiogenic factors [18]. Platelets must be activated to release the contents of alpha granules to the external milieu where they exert potent biological effects. In many *in vitro *or *in vivo *studies (or both), activation of PRP is accomplished by adding bovine or human thrombin with calcium chloride [8,21,22,25-28]. However, it would be more feasible for clinical use if the concern about immunogenic reactions or disease transmission in using xenogeneic or allogeneic blood products were eliminated. In addition, the use of thrombin was of concern to us because thrombin has been shown to degrade cartilage tissues [29] and to cleave the same site as plasmin [30,31]. Therefore, in this study, we used a mixture of autologous serum and calcium chloride for activation of PRP.

The administration of PRP-releasate into degenerated IVDs greatly restored the decrease in disc height following anular needle puncture in this *in vivo *rabbit model. In a previous study using the same animal model, the injection of OP-1 [11] or growth differentiation factor-5 (GDF-5) [24] induced restoration of disc height to a level approaching 90% of that of a normal non-punctured disc. The results of our study suggest that the administration of PRP-releasate had an effect on the structural restoration of disc height similar to those of OP-1 or GDF-5 or both.

The major advantage of using PRP over purified growth factors is that PRP can be isolated from an autologous source, thus eliminating concerns about the potential for cancer [32] or autoantibodies. In addition, the preparation of PRP can be performed in a regular hospital setting with a minimum requirement for equipment and less vigorous regulatory oversight. However, the inter-individual variability in the concentration of growth factors found in PRP, compared with the well-controlled recombinant growth factors currently in clinical trials (OP-1 and GDF-5), may result in inconsistent effects.

MRI has recently been used not only for morphological evaluation but also for characterization of the structural organization and matrix content of the IVD. The T2 mapping technique has shown its potential to quantitatively evaluate changes in the molecular composition and structural organization of the IVD [33-36]. In our study, the T2 mapping technique was used to detect and quantify changes in the matrix structure and integrity of degenerated rabbit IVDs injected with PBS and PPP- and PRP-releasate. Our results showed that the mean T2 value in the AF and NP region of the PRP group was not increased significantly over other groups, even though a minor increase was seen. Additional samples may be needed to show treatment effects on T2 values, which may have been subtle.

The histological analyses showed that the injection of PRP-releasate induced an increase in the number of chondrocyte-like cells in the NP and the anterior inner AF, and this suggests tissue phenotype changes from fibrotic tissue to cartilaginous tissue. No inflammatory reactions, such as invasion of inflammatory cells or microvessels (or both), or ossification within the IVD tissue was found, suggesting that PRP-releasate had no adverse effects on disc tissues.

In the radiographic and histological analyses, PPP-releasate also induced a reparative effect on degenerated IVDs, although its effect was less than those of the PRP-releasate. Previous *in vitro *studies [21,22] have shown that PPP has effects on cell proliferation and matrix metabolism similar to those of fetal bovine serum, which is the most widely used serum supplement for *in vitro *cell culture; this suggests that PPP also contains growth factors and has the potential to stimulate the matrix metabolism of degenerated IVDs.

In the past, two different groups have performed *in vivo *intradiscal injection studies by using PRP to evaluate its effect on disc degeneration in animal models. Chen and colleagues [8] reported the effect of PRP on disc degeneration both in an *ex vivo *organ culture system and an *in vivo *porcine disc degeneration model. The releasate isolated from clotted PRP, which was induced by addition of bovine thrombin, was injected into the degenerated disc induced by chymopapain. In their study, PRP, which was shown to promote NP regeneration, also resulted in the upregulation of chondrogenesis and extracellular matrix accumulation. In the *in vivo *model, the recovery of disc height was not significant (*P *= 0.5). This may be due to the use of thrombin. With consideration for a future clinical application, we have decided not to use thrombin, but rather autologous serum and calcium, to activate PRP.

The other group [37,38] reported *in vivo *intradiscal injection studies using PRP-impregnated biodegradable gelatin hydrogel microspheres (PRP-GHMs) in a rabbit disc degeneration model induced by the partial aspiration of the NP with a 21-gauge needle. Two weeks after the initial surgery, autologous PRP alone or PRP-GHMs were injected into the degenerated discs. Contrary to the results of our study, the progression of disc degeneration was significantly suppressed by the combined administration of PRP-GHMs but not by PRP alone. Possible explanations for the conflicting findings concerning the effect of PRP would be, first, the differing methods used in the processing the injectable agents. We injected PRP-releasate isolated from activated platelets into the degenerated disc, whereas in the study by Nagae and colleagues [37], the PRP alone injected into the degenerated disc was prepared without platelet activation (without coagulation). Indeed, in an article just published, DeLong and colleagues [39] state that the manner in which platelet activation occurs is a variable that determines the efficacy of PRP; they propose a classification system for PRP publications that includes 'platelet activation manner'. Second, differences in the disc degeneration animal models and evaluation methods could be reflected in the conflicting results. The anular puncture model has been shown to induce controlled and progressive disc degeneration and effectively detect the therapeutic effects [11,24,40]. A controlled degree of disc degeneration may be required to test the effects of biological therapies.

A limitation of this study is that the well-characterized rabbit anular puncture model [23] used is a short-term (1-month) injury animal model of disc degeneration. Although this model does not truly reflect the course of human disc degeneration, similar histological and biomechanical changes have been previously reported [11,23,24] and confirmed in this study. Therefore, the results of this study may contribute to the understanding of the cellular responses and the mechanism of the repair process after PRP application in degenerative discs.

## Conclusions

We have shown that the administration of autologous PRP-releasate was effective in restoring disc height and increasing chondrocytic cells in the IVD of the rabbit anular puncture disc degeneration model. Importantly, the PRP-releasate prepared in this study was isolated from autologous PRP, which was activated without using xenogeneic or allogeneic blood products. The results of this study showing that the application of autologous PRP-releasate is effective for IVD therapy could provide valuable information for the consideration of PRP for a clinical application.

## Abbreviations

AF: anulus fibrosus; ANOVA: analysis of variance; BMP: bone morphogenetic protein; DHI: disc height index; EGF: epidermal growth factor; GDF-5: growth differentiation factor-5; IGF-1: insulin-like growth factor-1; IL: interleukin; IVD: intervertebral disc; MRI: magnetic resonance imaging; NP: nucleus pulposus; OP-1: osteogenic protein-1; PBS: phosphate-buffered saline; PDGF: platelet-derived growth factor; PPP: platelet-poor plasma; PRP: platelet-rich plasma; PRP-GHM: platelet-rich plasma-impregnated biodegradable gelatin hydrogel microsphere; ROI: region of interest; TE: time-to-echo; TGF-β: transforming growth factor-β.

## Competing interests

The authors declare that they have no competing interests.

## Authors' contributions

SO helped to perform data acquisition and statistical analysis and write the manuscript. KA helped to perform data acquisition and statistical analysis and write the manuscript, conceived of this study, and made substantial contributions to the study design. KM contributed to the study design, interpreted the data, and finalized the manuscript. WB performed MRI data acquisition and interpreted the data. TI, RM, YA, and YK performed data acquisition. AU and AS contributed to the study design and coordination. All authors read and approved the final manuscript.
